# Antibodies against Phosphorylcholine—Implications for Chronic Inflammatory Diseases

**DOI:** 10.3390/metabo13060720

**Published:** 2023-06-01

**Authors:** Johan Frostegård

**Affiliations:** IMM, Nobels Väg 13, Karolinska Institutet, 17165 Stockholm, Sweden; johan.frostegard@ki.se

**Keywords:** antibodies, inflammation, SLE, atherosclerosis, immunity, phosphorylcholine

## Abstract

Atherosclerosis and its main consequence, cardiovascular disease (CVD) are nowadays regarded as chronic inflammatory disease conditions, and CVD is the main cause of death in the world. Other examples of chronic inflammation are rheumatic and other autoimmune conditions, but also diabetes, obesity, and even osteoarthritis among others. In addition, infectious diseases can have traits in common with these conditions. Systemic lupus erythematosus (SLE) is a prototypical autoimmune disease, where atherosclerosis is increased and the risk of CVD is very high. This is a clinical problem but could also shed light on the role of the immune system in atherosclerosis and CVD. Underlying mechanisms are of major interest and these are only partially known. Phosphorylcholine (PC) is a small lipid-related antigen, which is both a danger associated molecular pattern (DAMP), and a pathogen associated molecular pattern (PAMP). Antibodies against PC are ubiquitous and 5–10% of circulating IgM is IgM anti-PC. Anti-PC, especially IgM and IgG1 anti-PC, has been associated with protection in the chronic inflammatory conditions mentioned above, and develops during the first years of life, while being present at very low levels at birth. Animal experiments with immunization to raise anti-PC ameliorate atherosclerosis and other chronic inflammatory conditions. Potential mechanisms include anti-inflammatory, immune modulatory, clearance of dead cells and protection against infectious agents. An intriguing possibility is to raise anti-PC levels through immunization, to prevent and/or ameliorate chronic inflammation.

## 1. Introduction

Atherosclerosis and its consequence, cardiovascular disease (CVD) as myocardial infarction (MI) and stroke, are the major causes of death in the world. Atherosclerosis is nowadays regarded as a chronic inflammation and CVD could thus be described as a chronic inflammatory condition. This also applies to other major diseases, including rheumatic and autoimmune conditions, diabetes, obesity and others, including osteoarthritis and dementia [1].

Atherosclerosis is characterized by accumulation of dead cells and oxidized low density lipoprotein (OxLDL) in the intima of large- and middle-sized arteries, with interesting differences, for example arteries of the arm are less affected, if at all. Atherosclerosis could thus to some extent be described as a defective clearance of OxLDL and dead cells. Typical of atherosclerosis is also infiltration of immune competent cells, mostly macrophages, dendritic cells and other antigen presenting cells, and also activated T cells. These cells produce mainly proinflammatory cytokines. OxLDL can activate these cell types and also causes cell death and is taken up by macrophages which develop into inert foam cells which eventually die. OxLDL is thus one major candidate to be a cause of the disease [1,2,3]. In addition, calcification is a typical feature of atherosclerosis. Most likely this is—like atherosclerosis itself—a defence system which goes awry, when becoming chronic [1].

It is interesting to note that the inflammatory nature of atherosclerosis was described a long time ago, by famous pathologists from the nineteenth century, namely Rokitansky and Virchow who reported inflammation as a feature of atherosclerosis; however, they debated whether this inflammation was a primary phenomenon (Virchow) or secondary to other causative agents (Rokitansky) [4]. Interestingly, both were right, it could be claimed; for example, in autoimmune conditions, an increased risk of atherosclerosis and atherosclerotic plaques with ensuing CVD follows and atherosclerosis also develops in individuals who otherwise are healthy [1].

The concept that human atherosclerosis is an inflammatory condition was recently confirmed in the Cantos study, where IL-1 inhibition with a monoclonal antibody, canakinumab deceased major cardiovascular events [5]. Further, colchicine, an ancient medicine which has anti-inflammatory properties and is still used in goiters, had a positive effect on patients with CVD [6].

## 2. Phosphorylcholine as Danger and Pathogen-Associated Molecular Pattern

In atherosclerotic lesions, several damage-associated molecular patterns (DAMPs) are expressed on damaged and dead cells and also on OxLDL. These have a function to attract and activate the body’s defence systems, and can be proinflammatory or even dampen inflammation, depending on the context. Examples of DAMPs include malondialdehyde (MDA) and phosphorylcholine (PC), among others, which our research group has focused on, especially PC. Both have proinflammatory properties, when exposed on OxLDL and in other contexts (though not on apoptotic cells), and may thus contribute to OxLDL´s immune stimulatory and proinflammatory properties. Moreover, compounds such as cardiolipin (CL), especially oxidized CL and phosphatidylserine can function as DAMPs [1,7].

The present review is focused on PC, which is abundant as a membrane phospholipid where PC is the polar head group [8]. Phosphatidylcholine in cells and LDL can be modified oxidatively, chemically or by enzymes such as phospholipase A2 (PLA2). Oxidation of phosphatidylcholine is characterized by loss of fatty acids in the sn-2 position, which exposes PC and also has some proinflammatory effects which could promote low grade chronic inflammation [9]. It is thus important to emphasize that PC is not exposed to any significant degree in the normal artery, which occurs much more in arteries where atherosclerosis is developing.

OxLDL is raised in the circulation in hypertension, systemic lupus erythematosus (SLE) and other conditions, as determined by PC exposed on circulating LDL [10,11].

PC-exposing lipids have potentially atherogenic properties, by mediating vascular inflammation, apoptosis induction, endothelial dysfunction and endoplasmatic reticulum (ER)-stress [8,12,13]. In line with this are results from mouse models, where such compounds promote atherosclerosis [14].

In addition to being a DAMP, PC, in contrast to the other mentioned DAMPs, is also a pathogen associated molecular pattern (PAMP). It is well known that PC is exposed in the cell wall of Streptococcus pneumoniae and immunization with PC can ameliorate bacterial infection in animal models [15,16,17,18]. PC is also well known as an antigen exposed in other microorganisms including nematodes and helminths [19,20,21]. In line with this, we determined that infection with Treponema is strongly associated with anti-PC levels at Kitava, Papua New Guinea [1].

## 3. Anti-PC as Protection Marker in Chronic Inflammatory Conditions

In early studies, antibodies against OxLDL (anti-OxLDL) were reported as risk markers, associated with CVD and atherosclerosis [22,23,24,25,26]; however, we reported for the first time that antibodies (hereafter, anti-OxLDL) can be associated with protection in CVD [27]. These differences between studies could be related to the complexity of OxLDL, for example, anti-OxLDL has been reported to have properties similar to anti-phospholipid antibodies, which are thrombogenic, and thus a potential cause of CVD [28].

Since anti-OxLDL studies give conflicting results, we performed most of our research on components of OxLDL, especially PC and malondialdehyde (MDA). The focus in this review is PC.

We reported for the first time that anti-PC is a protection marker, IgM anti-PC being negatively associated with atherosclerosis progress in patients with hypertension and proposed that especially low levels means high risk, and also high levels being associated with low risk of disease [29]. After that we published more reports of IgM anti-PC as a marker for protection both in CVD, including stroke and MI, and atherosclerosis (including measures of intima–media thickness and progression), and low levels being associated with increased risk and/or high levels with decreased risk [1].

While we determined that patients with acute coronary syndrome (ACS) and low IgM anti-PC have a worse prognosis than those with high levels, another group did not confirm this [1,30,31]. However, in our study, the associations were relatively weak and significant only at the lowest levels of IgM anti-PC and in acute disease, other factors including acute inflammatory and other effects, such as consumption of anti-PC into damaged tissue and lesions could play a role. The role of anti-PC in acutely sick individuals is not clear and needs further study.

Our findings in Alzheimer´s disease in relation to anti-PC are more complicated. We conducted a nested case-control study of incident dementia cases (serum collected before onset of dementia) matched to controls and also a case-control study of prevalent dementia cases with matched controls from the Swedish Twin Registry. Even though patients with dementia had significantly lower IgM anti-PC levels, there was no association with incident cases, in contrast to our findings in relation to incident CVD cases [1]. Therefore, this speaks against anti-PC playing a causative role in Alzheimer´s disease, though it may be useful in relation to diagnostics.

We have also studied other chronic inflammatory and/or autoimmune conditions, given the associations published for IgM anti-PC. Of special interest are conditions with increased risk of atherosclerosis and CVD, since this could give insights into immunological aspects of atherosclerosis as a kind of “human model”, in addition to being of importance for the diseases and their complications per se.

A strong and significant protective association between mortality and anti-PC was noted among patients with severe chronic kidney disease (CKD) where the risk of CVD is high. This is the case also among individuals with rheumatic diseases. One example is rheumatoid arthritis (RA) where IgM anti-PC is associated with protection against CVD in, and also with prevalence of atherosclerotic plaques after 5 years. In addition, IgM anti-PC is associated with being a non-responder to biologics (TNF-inhibitors) in RA [1].

Interestingly, other groups have reported associations with protection in other rheumatology-related conditions such as osteoarthritis [32] and vasculitis [33] and also in relation to upper respiratory infections (which is interesting given PC’s role as both a DAMP and PAMP [34].

We demonstrated that in SLE, anti-PC is significantly associated with protection from CVD and also in relation to prevalence of atherosclerotic plaques and other disease manifestations. In other systemic inflammatory diseases, such as Sjögren´s syndrome and Systemic sclerosis, there is also a significant association with anti-PC as a marker of protection [1,35,36,37].

Studies from other groups have largely confirmed and extended this notion and associations with protection have been reported for inflammation-related diseases as different as vasculitis and osteoarthritis [29,38,39,40,41,42,43]. Moreover, our findings in SLE have been largely confirmed by other researchers [38,39,40,41,42,43,44].

In Systemic lupus erythematosus (SLE), the risk of CVD is very high as compared to age- and sex-matched controls [11,45,46]. Therefore, SLE is one focus of this review. Atherosclerosis, especially the prevalence of atherosclerotic plaques, is significantly increased in SLE as compared to age- and sex-matched controls [11,47,48]. Additionally, the prevalence of echolucent plaques which may represent vulnerability, is increased in SLE [35,49,50]. Another example of an autoimmune and chronic inflammatory disease which is related to both CVD and neurological complications is thyroiditis [51].

About 5–10% of the circulating IgM pool may be IgM anti-PC and we have detected IgM anti-PC in all sera or plasma tested so far [1]. We have also studied other isotypes and subclasses of anti-PC in relation to prediction and outcome. We have not been able to determine IgG3 and IgG4 anti-PC at significant levels and have focused on IgG1 and IgG2 anti-PC. In our studies, IgG1 anti-PC is a stronger protection marker than IgG2 anti-PC. Further, IgG anti-PC in our studies is not as good as IgM anti-PC as a protection marker [52].

The protective properties of anti-PC are mainly present in IgM and IgG1 [36,52]. In line with this, IgM and IgG1, but not so much IgG and IgG2 anti-PC are associated with less atherosclerosis progress, vulnerable plaques and mortality in CKD [53]. IgA is mainly of importance at mucosal surfaces and IgA anti-PC is likely to be of less importance in circulation and CVD. Further, IgA anti-PC is much less studied. We are not aware of studies on IgD or IgE anti-PC. IgG2 anti-PC has bactericidal properties and recognizes capsulated bacteria, with carbohydrate antigens [54,55]. This may provide an explanation to why the risk of CVD is raised in periodontitis, in spite of increased IgG2 anti-PC levels [56,57].

Anti-PC were described in the 1970s, as PC-binding myeloma proteins [58]. They are usually described as natural antibodies and germ-line encoded also in humans, which is the case with mice, where one clone, TI5 dominates. However, human anti-PC undergo somatic mutation with Ig switch. Furthermore, and in line with this, they are T cell dependent [59]. We also reported that humans are born with very low levels of anti-PC which slowly increases, but are not at par with their mothers´ levels even at 2 years of age. It is therefore likely that the environment plays a role, especially the microbiome. More research is needed to delineate which bacteria or even virus can promote anti-PC development [60].

Brown bears (Ursus arctos) could also shed an unexpected light on atherosclerosis, CVD and chronic inflammation in general, but also on environmental influence on anti-PC levels. These animals are immobilized and uremic during hibernation, for about half a year, and also have very high blood lipid levels. In spite of this, they show no signs of atherosclerosis at all, and therefore appear to be strongly protected against atherosclerosis and CVD during hibernation. They develop very high levels of IgM and IgG1 anti-PC during winter, which may even be a natural immunization against atherosclerosis [61].

In line with this, the genetic contribution of IgM anti-PC is relatively modest. The heritability was 0.4 and anti-PC levels were determined to be partly influenced by dominance genetics and also independent of other CVD biomarkers. We also studied the genetics of anti-PC by combining large-scale studies where we had measured anti-PC and also had genetic information. From our genome-wide association studies (GWASs) in four cohorts, six single nucleotide polymorphisms (SNPs) were discovered and replicated to be associated with IgM anti-PC. The leading SNP is known to be the top risk allele for chronic lymphocytic leukemia (CLL) but only explained 0.6% of CLL variance. Nevertheless, anti-PC was associated with protection against CLL [62]. Clearly more studies are needed to determine if anti-PC may play a role in cancer development or prevention. After all, associations between increased cancer risk and inflammation have been known for a long time. Associations of anti-PC with different disease conditions are summarized in Table 1.

## 4. SLE, Atherosclerosis and CVD

SLE is of special interest in the context of PC and anti-PC. It is relatively rare and often considered as a prototypic autoimmune disease mainly affecting women (above 90%). It is important to note that SLE shows a huge variation in symptoms which could vary between mild disease and life threatening disease with severe autoimmune damage of inner organs as kidneys. There is considerable variation in incidence, which may reflect both diagnostic difficulties and underlying genetic and environmental differences. In a meta-analysis from 2017, incidence from 0.3/100,000 person years to 23.2/100,000 person years has been reported in different studies with varying origins [63]. SLE varies much in disease manifestations, which could be severe, with engagement of inner organs as kidneys, heart and lung or could be more mild, with synovitis and typical symptoms from the skin. Immune complexes play an important role, as in nephritis. Typical of SLE are different types of autoantibodies, and diagnosis requires anti-nuclear antibodies to be settled, in addition to clinical manifestations. Due to the heterogeneity, diagnosis is often complicated, especially at early stages. The prognosis has improved after the introduction of anti-inflammatory medications as corticosteroids and hydroxychloroquine and recently biological treatments have been introduced, though they have not so far had the same type of success as biologics in rheumatoid arthritis or psoriatic arthritis and related conditions [64]. Even though the prognosis since the introduction of corticosteroids and other treatment modalities has improved considerably, there is still an increased mortality in SLE, with complications, especially lupus nephritis being important factors. Cardiovascular disease (CVD) could also be seen as another complication of the disease [64,65].

Another interesting feature of SLE not least in relation to CVD is anti-phospholipid antibodies. These cause arterial and venous thrombosis by different mechanisms, including a direct inflammatory and prothrombotic effect on the endothelium; another example is interference with the coagulation system by inhibiting binding of protective compounds, promoting atherosclerotic complications in SLE. This anti-phospholipid antibody syndrome can also be primary, e.g., be present without an SLE diagnosis [46].

As in rheumatic diseases in general, diagnostic criteria are used, since the causes of the diseases mostly remain obscure. The European League Against Rheumatism/American College of Rheumatology published the most recent ones in 2019, where positive anti-nuclear antibodies (ANA) was required as an entry criterion; clinical and immunological manifestations were combined for diagnosis (over a certain level) [66]. As the presence of ANA illustrates, nuclear material, likely from dead cells and immune reactions against it, is of major importance for the disease development [67]. Another related typical feature of SLE is the defective clearance of dead cells [68].

Furthermore, there are other immunological imbalances in SLE, where a lower proportion of T regulatory cells (T regs) is common. T regs are known to suppress autoimmune immune reactions, among other functions [69,70,71]. Other potential underlying immunological causes which have been discussed include elevated type I interferons (IFNs), where gain-of-function-variants are related to SLE. [72,73]. Neutrophil extracellular traps (NETs) are also enhanced in SLE and may play a role in activating IFN pathway activation [74]. Furthermore, genetic, epigenetic and environmental factors have been discussed in this context [46,64,75,76,77,78,79]. There is also variation in estimations of mortality in SLE, but a recent meta-analysis indicates that this is still raised by two and six tenths times in spite of improved therapy. The major causes of death are renal damage, CVD, and infection [80]. Other studies report that mortality is increased by two to three times [63,81,82,83,84]. After improvement in treatment with cortisone, hydroxychloroquine and other medications, later complications, especially CVD came in focus. In an early study, a bimodal pattern of SLE was described, where early manifestations of SLE were followed by CVD later on [85].

In addition, there are variations here and a very high risk (fifty-fold) has been reported for myocardial infarction (MI) in SLE compared to the general population [45]. In general, a combination of traditional and non-traditional risk factors have been described [86,87,88,89,90,91,92,93,94,95]. In the first study with population-based controls, we reported that non-traditional risk factors include the level of OxLDL in the circulation (as determined by the expression of PC on LDL), and also anti-phospholipid antibodies and cortisone use. Among traditional risk factors, dyslipidemia (though not raised LDL) was prominent. Other traditional risk factors such as smoking, blood pressure and diabetes were not associated with CVD. Both cortisone treatment and osteoporosis were associated with CVD [11]. In recent studies, similar results have been published, with some variation and attention should be paid to prevention and treatable traditional and non-traditional risk factors for CVD in SLE [95,96,97,98,99,100,101,102,103,104].

The high risk of CVD in SLE is an important clinical problem, but could also shed light on inflammatory and immune mechanisms in CVD and atherosclerosis. Interestingly, accumulation of dead cells is a major feature in atherosclerosis where clearance of dead cells is thus dysfunctional, which is also the case in SLE. Another compound, believed to be of major importance in atherosclerosis, OxLDL and accumulates in plaques [1] is increased in SLE where it is also associated with CVD [10]. SLE, like atherosclerosis is a chronic inflammatory condition. Refs. [1,2] in both conditions as discussed above, show that anti-PC is associated with protection, e.g., in progress of atherosclerosis in hypertension and in SLE, there is an association with protection against atherosclerosis, especially atherosclerotic plaques.

In general, atherosclerosis, by use of different measurements, is increased in SLE [105], where it is associated with both traditional and non-traditional risk factors [11,35,106,107,108,109,110,111,112]. Most likely, the increased atherosclerosis in SLE is more related to an increase in the amount (and potentially vulnerability) of plaques than a general increase in intima–media thickness, which is a common measure of atherosclerosis. Another common feature of atherosclerosis and SLE is a proinflammatory T cell profile, with a relative decrease in T regs [2,113,114,115,116,117,118]. Moreover, anti-PC promotes proliferation of T regs in T cells from atherosclerotic plaques and from SLE patients [71].

## 5. Animal Models and Immunization with PC

Passive immunization with anti-PC was demonstrated to inhibit meningitis caused by Streptococcus pneumoniae in mouse models in the 1980s [16]. Active PC-immunization, raising anti-PC levels three-fold was demonstrated to reduce atherosclerotic aorta root lesions by >40% in the PC-immunized mice (*p* < 0.01). Here, the apoE k/o models were used, and as a protein carrier, keyhole limpet hemocyanin was used. In addition, foam cell formation was reduced [119]. In another paper published in Nature Medicine, PC itself was not used as an antigen, but instead Streptococcus pneumoniae, in another atherosclerosis model, Ldlr(-/-) mice. This induced antibodies against OxLDL, but also against PC and ameliorated atherosclerosis significantly [120]. In a recent paper, a nasal double DNA adjuvant system with PC-KLH induced IgM anti-PC and reduction in aortic atherosclerosis in apoE k/o mice [121]. Additionally, passive immunization by IgM anti-PC reduced atherosclerosis in apoE ko mice using a venous graft model and weekly intraperitoneal injections of anti-PC. The vein graft size was significantly reduced, and also the inflammatory content of lesions [122]. In another recent study, a femoral artery cuff model in ApoE3*Leiden mice was implemented, to mimic atherosclerosis complications in humans. Here, immunization with an IgG1 monoclonal antibody significantly reduced vascular inflammation and accelerated atherosclerosis, also reducing inflammation and OxLDL-uptake [123]. Interestingly, inflammatory arthritis in a mouse model was ameliorated by active immunization with PC [124] and likewise, immunization ameliorated SLE [125].

## 6. Potential Underlying Mechanisms

Several different mechanisms have been described, which could explain the clinical associations with protection and also the findings in animal models.

IgG anti-PC (IgM was not studied) inhibits pro-inflammatory effects by PC exposing proinflammatory phospholipids on endothelial cells [126]. Such PC-exposing lipids are raised in SLE and associated with CVD in SLE, and also raised in hypertension.

Another immunomodulatory and anti-inflammatory property is the promotion of T cells to develop into T regulatory cells, which was demonstrated in ex vivo systems using cells from different sources such as healthy donors, atherosclerotic plaques, and SLE-patients [71]. Another mechanism which could be equally important is the inhibition of uptake of OxLDL in macrophages in the arterial wall, which develop into inert foam cells which eventually die [127]. Cell death and lack of clearance of dead cells is a major feature of atherosclerosis, and, interestingly, also of SLE. One mechanism by which anti-PC could be protective is by inhibiting the cytotoxic effects of lysophosphatidylcholine, which is generated during LDL-oxidation. In our opinion, the most important atheroprotective effect of IgM and IgG1 anti-PC is its ability to increase clearance of dead cells [36,128]. This could also be of major importance in SLE [1].

## 7. Old Friends/Hygiene Hypothesis and Anti-PC

In Kitava, Papua New Guinea, the prevalence of CVD and chronic inflammatory conditions is very low [129]. We therefore studied anti-PC in this population, and reported that IgM, IgG and IgA anti-PC IgM are decreased among Swedes compared with age- and sex-matched individuals from Kitava. We have suggested that this could contribute to the low prevalence of these conditions and that the high anti-PC levels could be related to the infectious panorama since many microorganisms expose PC, one example being Trepanoma infections, leading to Jaws [1]. PC is exposed in other types of microorganisms as nematodes and helminths, Refs. [19,20,21] and sixty-nine proteins with the PC-epitope have been identified in this context [21].

In line with this, ES-62, which is a nematode protein with huge amounts of PC, decreased atherosclerosis development and ameliorated SLE in a mouse model, with a concomitant rise in anti-PC [125].

According to the Old Friends/hygiene-hypothesis, lack of exposure to environmental agents especially infections could contribute to autoimmune and allergic diseases in Western countries [130,131,132,133]. We therefore proposed a development of this hypothesis, where lack of exposure to PC exposing microorganisms could contribute to chronic inflammatory diseases [1].

## 8. Conclusions

Anti-PC, especially IgM and IgG1 anti-PC, are associated with protection in chronic inflammatory disease conditions, especially atherosclerosis and CVD, but also SLE and CKD. In SLE these associations are strong and interesting both from a clinical and a more mechanistic point of view. In a growing number of other chronic inflammatory conditions, similar associations are described. This is supported by animal experiments and plausible underlying mechanisms have been described.

## 9. Future Directions

The role of anti-PC and its subclasses and isotypes to determine the risk of future atherosclerosis and CVD ought to be determined in larger cohorts, in order to confirm and also define in more detail what role it could play as a new independent risk marker. While studies on atherosclerosis and CVD are relatively many nowadays, larger studies, perhaps in combination with large-scale studies on medications are warranted. Studies on rheumatic diseases and anti-PC are less prevalent, and confirmation in larger ones are of interest. Especially osteoarthritis where only one study is available, and where knowledge of disease mechanisms is scarce would be of great interest, also since there are no medications available. Likewise, in cancer, only one study exists suggesting an association between anti-PC and incident disease, and here studies on different types of cancer would be of major importance. Anti-PC seems less promising in dementia, especially Alzheimer´s disease, even though other types of dementia including vascular dementia and other chronic brain or neurologic conditions have not been studied.

An interesting possibility is to increase anti-PC levels by immunization, especially IgM and IgG1 anti-PC, which could be of interest in prevention and therapy. Such immunization is supported by several animal experiments. Depending on findings from further studies on anti-PC as a risk marker, specific diseases could be targeted in larger clinical trials, where a common denominator in today´s disease panorama, chronic inflammation, is in focus. Such a PC-vaccine could be combined with diagnostic determination of anti-PC levels in a theragnostic concept of personalized medicine, where those with low levels of anti-PC are most eligible for vaccination, even though it is likely that higher levels are beneficial in general.

## Figures and Tables

**Table 1:** Protective properties of IgG1 and IgM anti-PC in different disease conditions *.

Atherosclerosis	Atherosclerosis progress during 4 years among hypertensives. (IgM) [29]
Myocardial infarction and stroke	Incident cases (IgM [1] and IgG1 [52])
Rheumatoid arthritis	Cross sectional and prospectively (IgM), Cross sectional (IgG1 [1])
Systemic lupus erythematosus	Cross sectional and prospective: SLE manifestations, CVD and atherosclerosis (IgM [36,52])Cross sectional, SLE manifestations and CVD (IgG1 [52])
Sjögren´s syndrome	Cross sectional (IgM) [36]
Systemic sclerosis	Cross sectional (IgM) [36]
ANCA-associated vasculitis	Cross sectional (IgM) [33]
Osteoarthritis	Cross sectional (IgM) [32]
Chronic kidney disease	Mortality, incident CVD and atherosclerosis progress. (IgM and IgG1 [53]
Alzheimer´s disease	Prevalent cases, cross sectional (IgM) [1]
Upper respiratory infections	Cross sectional (IgM) [34]
Chronic lymphatic leukemia	Incident cases (IgM) [62]

* In general, IgG and IgG2 anti-PC are less associated with protection. References either from review or from first report.
